# The Role of Xanthine Oxidase in Pregnancy Complications: A Systematic Review

**DOI:** 10.3390/antiox13101234

**Published:** 2024-10-14

**Authors:** Lorenzo Annesi, Giovanni Tossetta, Claudio Borghi, Federica Piani

**Affiliations:** 1 Hypertension and Cardiovascular Risk Research Center, Medical and Surgical Sciences Department, Alma Mater Studiorum University of Bologna, 40138 Bologna, Italy; lorenzo.annesi@studio.unibo.it (L.A.); claudio.borghi@unibo.it (C.B.); 2 Department of Experimental and Clinical Medicine, Università Politecnica delle Marche, 60126 Ancona, Italy; g.tossetta@pm.univpm.it

**Keywords:** xanthine oxidoreductase, xanthine dehydrogenase, xanthine oxidase (XO), oxidative stress, placenta, pregnancy, pregnancy complications, preeclampsia, gestational diabetes

## Abstract

Xanthine oxidoreductase (XOR) is an enzyme involved in the oxidation of hypoxanthine and xanthine to uric acid. XOR has two isoforms: xanthine dehydrogenase and xanthine oxidase (XO). XO plays a major role in oxidative stress, causing the formation of reactive oxygen species. In the present study, we aimed to summarize the evidence on the association between XO and pregnancy complications. The PRISMA checklist guided the reporting of the data. We conducted systematic searches in the PubMed and Web of Science databases to identify all human studies investigating XO in pregnancy diseases up to June 2024. A total of 195 references have been identified and 14 studies were included. Most studies focused on women with PE and GD. Overall, all the included studies found a statistically significant increase in maternal, placental, and/or fetal XO levels, activity, or tissue expression in women with pregnancy complications, compared to those with uncomplicated pregnancies. Although promising, the quality and dimension of the included studies do not allow for a definitive answer to the question of whether XO may play a crucial role in pregnancy complications. Future studies are warranted to confirm if XO could represent a prognostic and therapeutic marker in pregnancy complications and their impact on long-term maternal and offspring cardiovascular health.

## 1. Introduction

Pregnancy is a metabolic state characterized by an elevated energy demand and an increase in oxygen consumption, which results in high mitochondrial activity and the production of many reactive oxygen species (ROS) in the placenta [1]. ROS are crucial signal transducers in physiological processes of normal placentation, including proliferation, migration, and angiogenesis, but ROS overproduction and the depletion of antioxidant systems can lead to oxidative stress (OS) and abnormal development of the placental vessels, leading to placental insufficiency [1,2,3]. ROS play a crucial role in cellular signaling, but their excess causes cellular damage, lipid peroxidation, DNA oxidation, protein modifications, and, consequently, cellular and tissue dysfunction [4]. Therefore, a dynamic balance between the generation of ROS and the actions of antioxidant systems (including enzymatic systems such as xanthine oxidoreductase (XOR), superoxide dismutase (SOD), glutathione peroxidase, glutathione reductase and catalase, and nonenzymatic antioxidants such as vitamin C and E, beta carotene, selenium, zinc, taurine, and manganese) is essential for maintaining ROS at optimal levels for proper cellular functioning [2,3]. XOR belongs to the highly conserved family of molybdo-flavoenzymes and performs the last two steps of purine catabolism [5]. XOR catalyzes the oxidation of hypoxanthine and xanthine to uric acid, which is the end product of purine catabolism in humans [6]. This enzyme has two forms that coexist in vivo: xanthine dehydrogenase (XDH) and xanthine oxidase (XO). XDH requires nicotinamide adenine dinucleotide (NAD+) and produces uric acid and reduced nicotinamide adenine dinucleotide (NADH). XO requires molecular oxygen as an electron acceptor and generates uric acid and ROS, namely superoxide ion (O^2•−^) and hydrogen peroxide (H_2_O_2_), if it catalyzes the monovalent or divalent electron transfer to O_2_, respectively. Under physiological conditions, the enzyme XOR exists mostly as XDH and is located in the cytosol and peroxisomes, and is also found in extracellular compartments, such as blood and milk, as a result of physiological cell turnover and of the apocrine secretion from the breast during lactation [5]. Under certain conditions, including ischemia and hypoxia, the enzyme XOR is released from the cell into the bloodstream and the ATP depletion triggers the reversible conversion of the dehydrogenase form to XO through the oxidation of a sulfhydryl group. When reperfusion and re-exposure to normal oxygen tension occurs, the increased XO activity and expression results in excessive production of ROS [5,7] Therefore, XO plays an important role in the OS-related tissue injury in all the diseases characterized by ischemia–reperfusion (H/R) conditions, including myocardial infarction, stroke, acute renal failure, and diseases affecting the maternal–fetal unit [7,8]. Indeed, XO may play a key role in many obstetric conditions characterized by altered placentation due to but not limited to insufficient trophoblastic remodeling of uterine spiral arteries and consequent increase in placental vascular resistance. The increased resistance and altered flow lead to an ischemic injury at the intervillous space that could trigger the conversion of XOR to XO, with increased OS contributing to the altered maternal adaptation to pregnancy and the onset of pregnancy-related pathological conditions [9]. XOR also plays a central role in redox homeostasis and adaptation to pregnancy by regulating uric acid levels. Indeed, uric acid is among the main antioxidant systems, accounting for 50% of the total antioxidant capacity of biological fluids in humans while, when present in the cytosol of the cells or an acid/hydrophobic environment, turns into a pro-oxidant agent, promoting OS [10]. Uric acid can scavenge oxygen radicals and peroxyl radicals in the hydrophilic environment, such us plasma, but loses the ability to break the radical chain propagation and can also form free cytotoxic radicals, such as aminocarbonyl, in a variety of systems within lipid membranes. Therefore, the double oxidant and antioxidant function of uric acid plays a key role in the redox homeostatic system, and an imbalance towards excess uric acid represents a pathophysiological mechanism in the onset of many diseases [11]. ROS-induced OS not only leads to important pregnancy diseases such as preeclampsia (PE) and gestational diabetes (GD) but could also alter fetal development [3]. Furthermore, OS increases the risk for the development of several chronic diseases later in the life of the offspring (including ischemic heart disease and type 2 diabetes mellitus) through epigenetic modifications due to harmful uterine-environmental exposures. On the other hand, in the mothers, excessive OS may influence fertility, by inducing apoptosis in oocytes and interfering with chromosomal disjunction during meiosis, although the presence of ROS is needed for hormone production and ovulation [9]. Prolonged exposure to high ROS concentration also contributes to a wide range of pathologies, such as cancers, cardiovascular diseases, and neurological diseases [12].

Major pregnancy complications are associated with an increased long-term cardiovascular risk for both the mother and the fetus; for instance, PE determines a 4-fold increased risk of future heart failure and a 2-fold increased risk of coronary heart disease and stroke [13], whereas GD is responsible for a 2.3-fold increased risk of cardiovascular events in the first decade postpartum [14]. These associations may be partly explained by XO-induced OS. Indeed, studies of large cohorts of patients have demonstrated that XO is independently associated with cardiovascular risk due to the detrimental systemic effects of OS. Excessive XO activity contributes to inflammatory responses, the oxidation of low-density lipoprotein particles, and the formation of advanced glycation end products, all associated with the atherosclerotic process and the development of major cardiovascular events. XO-derived OS may also contribute to the progression of cardiovascular disease-associated endothelial dysfunction since superoxide radicals inactivate endothelial NO, an important vasodilator agent [15,16,17]. Finally, the terminal product of the reaction catalyzed by XO is uric acid, which is increased both in some pregnancy pathologies and in cardiovascular disease [18,19].

In summary, XOR, and particularly, XO are involved in multiple pathophysiological mechanisms, such as OS, hyperuricemia, and H/R lesions, that, in turn, are associated with short- and long-term detrimental effects of pregnancy complications in both the mothers and their offspring. In the current study, we aimed to systematically review current evidence on the role of XO in pregnancy complications.

Although OS is a recognized mechanism contributing to preterm birth (PTB) and recurrent pregnancy loss (RPL), no studies investigating XO as a marker of OS in the context of RPL and PTB were identified. Therefore, we focused on the role of XO in HDP (HDP), GD, and intrauterine growth restriction (IUGR).

## 2. Materials and Methods

This systematic review did not require ethics approval because it involved completed research findings. The PRISMA checklist guided the reporting of this systematic review. We performed a systematic search using the electronic databases PUBMED and Web of Science. Two independent reviewers screened the articles and evaluated the quality of the included studies. Disagreements have been resolved by consensus or, when an agreement was not reached, with the help of a third reviewer.

### 2.1. Protocol

This systematic review was conducted according to the last Preferred Reporting Items for Systematic Reviews and Meta-analyses protocols (PRISMA-P) guidelines [20]. The systematic review was initially conducted as part of an internal project within the institution, and there was no requirement to register the protocol. To ensure transparency in the process, we intended to register the protocol, but data extraction and analysis were already completed.

### 2.2. Eligibility Criteria

The literature search was limited to the English language and human subjects. We excluded reviews and we included cross-sectional studies, case–control studies, cohort studies, case reports, case series, and histological studies. Exclusion criteria were as follows: publications evaluating markers of OS that have not assessed XO levels, activity, or expression; publications concerning the assessment of XO in diseases other than pregnancy complications; animal studies; and publications not in English. The eligible studies should include at least one of the following outcomes: miscarriage, IUGR, PE, gestational hypertension, premature delivery, and GD. Publications were included if maternal serum or cord blood XO levels, maternal blood, cord blood or placental XO activity, or XO expression in placental, or other tissue samples were assessed. No restrictions were made regarding the comparison groups or the XO measurement methods.

### 2.3. Information Sources and Search Terms

A systematic literature search was performed in the following databases from inception until 31 July 2024: Pubmed and Web of Science Core Collection.

Search terms used in the current search included: “Pregnancy”, “Xanthine oxidoreductase”, “Xanthine dehydrogenase”, “Xanthine oxidase”, “oxidative stress”, “placenta”, “pregnancy”, “early miscarriage”, “intrauterine growth restriction”, “preeclampsia”, “gestational hypertension”, “premature delivery”, and “gestational diabetes”.

### 2.4. Study Selection, Data Extraction and Quality Assessment

All articles were imported into Zotero. After eliminating duplicates, two reviewers independently screened the abstracts and full texts of the records to assess eligibility. Detailed descriptions of the participants and interventions in each study are provided in the text and summarized in Table 1.

The methodological quality and risk of bias of the selected studies were assessed using the STROBE standard [35], specifically by applying the 22-item STROBE checklist. Two authors independently performed the scoring, with any disagreements resolved through discussion and consensus. When agreement was not reached, a third author was consulted. Studies meeting 10 to 16 out of 22 criteria were classified as having medium quality and medium risk of bias; those exceeding or falling below this range were classified as high-quality and low-quality, respectively. The overall quality of the included studies was moderate to high, and the number of cases was low to moderate (the number of enrolled women, both cases and controls, was <50 in 7 studies, 50–99 in 4 studies, and 100–200 in 3 studies).

## 3. Results

**Figure 1:**
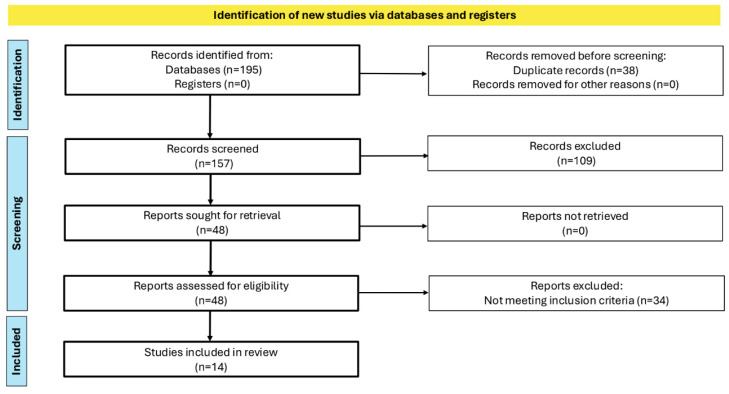
Flow diagram illustrating the article selection process of this review. XO: xanthine oxidase.

A total of 195 references were identified (107 from Pubmed and 88 from Web of Science). After removing duplicated articles (n = 38), 157 articles were screened. In total, 109 screened articles were excluded: 14 studies were removed because they were secondary studies and 95 studies were removed for not assessing XO with any kind of method. A total of 48 articles were eligible for full-text reading; 34 records of these were excluded from this systematic review for not assessing the relationship between XO and pregnancy complications listed in the eligibility criteria. In total, 14 articles fulfilled the inclusion criteria (Figure 1). No articles were found for the following outcomes: early miscarriage and premature delivery.

## 4. Discussion

**Figure 2:**
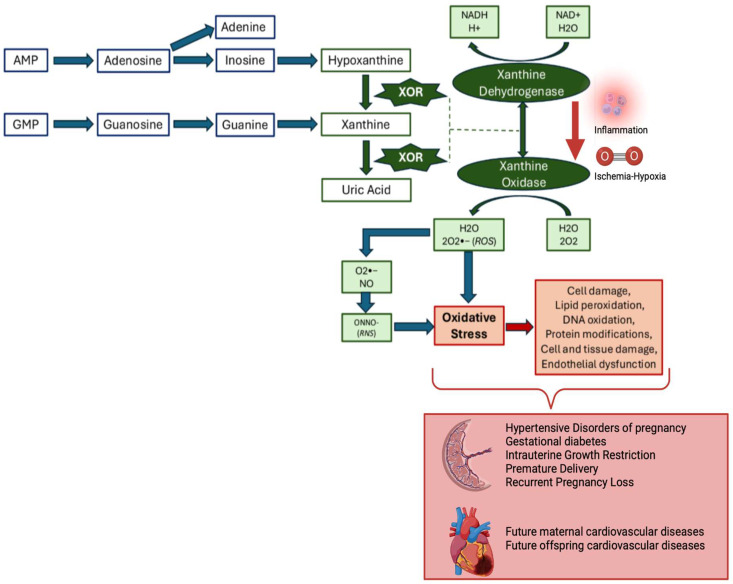
Catabolism of purines up to the formation of uric acid by the two isoforms of the enzyme XOR: xanthine dehydrogenase and xanthine oxidase. In case of ischemia, hypoxia, or inflammation, the conversion of the xanthine dehydrogenase into the xanthine oxidase isoform causes the excessive formation of superoxide radicals that increase OS. Furthermore, the superoxide radical (O^2−•^) reacts with the nitric oxide produced by the endothelium, determining the formation of peroxynitrite (ONNO-), which also contributes to OS. Then, OS causes damage at the subcellular, cellular, and tissue levels, inducing pregnancy complications and long-term cardiovascular dysfunction in both the mother and the offspring. List of abbreviations: OS, oxidative stress; XOR, xanthine oxidoreductase; ROS, reactive oxygen species; RNS, reactive nitrogen species. Image created with BioRender.com.

Figure 2 summarizes the metabolism of purines up to the formation of uric acid (terminal product in humans) and highlights the formation of superoxide radicals in the reaction catalyzed by XO. In conditions of ischemia, the conversion of XDH to XO promotes OS and, consequently, damages at the subcellular, cellular, and tissue levels. In the upcoming text, we aim to provide a summary of the current literature on the role of XO activity and expression, both at the placental and systemic levels, in the main complications of pregnancy.

XO-induced and XO-independent ROS are crucial signal transducers in the physiological processes of normal placentation, including proliferation, migration, and angiogenesis. Appropriate development of the placental vascular network requires vasculogenesis, angiogenesis, and trophoblast-mediated arterial remodeling [2]. In the first trimester, the embryo develops in a low-oxygen environment, allowing the proliferation of trophoblastic cells [36]. The trophoblastic invasion of uterine arteries converts the spiral arteries into a chamber of high flow and low velocity, resulting in higher oxygen tension in the intervillous space. At the beginning of the second trimester, the increase in oxygen tension and the high energy demand lead to increased mitochondrial activity, resulting in intrauterine mild ROS production, as a physiological response to the pregnancy [1]. As shown in Figure 2, when the production of ROS during pregnancy is not balanced by antioxidant defenses, the consequential OS could disturb fetal development and increase the risk for IUGR, PE, premature delivery, and GD [9]. Therefore, reducing OS during gestation may represent a therapeutic target to improve pregnancy outcomes. OS can be measured in three major ways: the direct measurement of ROS levels; the indirect measurement of ROS-induced protein, lipid, and DNA damage or ROS-producing enzymes; and the assessment of antioxidant status [37]. However, there is no consensus on which marker is the best in various obstetric conditions [38]. Assessing XO activity has advantages over other OS markers; it is involved in multiple pathophysiologic pathways and could provide an estimate of future cardiovascular risk since increased XO levels characterize both pathological pregnancies and cardiovascular diseases [13,14,17]. Furthermore, since XO inhibitors exert cardioprotective effects in patients with symptomatic hyperuricemia, they may exert similar benefits in women with a previous history of pregnancy complications [39]. Furthermore, the use of XO inhibitors has shown potential in reducing fetal brain damage due to asphyxia [40,41,42].

Before discussing the role of XO in specific obstetric pathological conditions, in the following, we summarize the general aspects of XO activity in humans, including the biochemical mechanisms underlying XO-induced ROS production, the methods used to assess XO activity, and the pathophysiological effects of XO activity dysregulation.

### 4.1. History, Molecular Characteristics, and Tissue Expression of XOR

XOR is a dimeric metallo-flavoprotein enzyme that catalyzes the final two steps of purine degradation: the oxidation of hypoxanthine and xanthine to uric acid. This enzyme consists of two subunits, each of 145 kDa: one contains a molybdopterin cofactor (Mo-co), where purine oxidation occurs, and the other contains a FAD cofactor, which is required for the oxidation of nicotinamide adenine dinucleotide (NAD+) and the reduction in molecular oxygen [43]. After human XDH cDNA was cloned and sequenced, and the relevant gene was located on chromosome 2 at the p22 band using the FISH method (fluorescence in situ hybridization) in 1994, studies were conducted to determine which tissue exhibited the highest enzyme expression [44,45]. A study published in 1998 quantified XOR gene expression by measuring the amount of RNA in tissue samples using RT-PCR (Reverse Transcriptase Polymerase Chain Reaction). XOR mRNA expression was found to be highest in the gut and liver, while the cardiac muscle and brain showed the lowest expression levels [7]. XOR is also detectable in other tissues and cell types, such as the kidney, lactating mammary gland, epithelial cells, and vascular endothelial cells. In many other tissues, XOR gene expression is low due to downregulation. Indeed, although XOR is present in all human tissues, in most cases the XOR gene is typically repressed at the transcriptional level [43]. However, various factors can upregulate XOR gene transcription, including hypoxia, inflammatory cytokines, and certain hormones [46]. For example, XOR expression increases during pregnancy due to exposure to glucocorticoids and prolactin [47].

The history of the discovery of XOR at the molecular and genetic levels, along with a more in-depth discussion of the tissues that express XOR, is summarized in the review by Kooij et al., 1994 [48]. The first studies on placental expression of XOR, conducted around the 1980s, reported the virtual absence of enzyme activity in fresh human placenta, measured spectrophotometrically [49]. However, using more sensitive laboratory methods, Many et al. in 1996 were the first to demonstrate the presence of XOR in the human placenta. They detected XDH/XO-RNA using Northern hybridization, and XOR tissue expression by immunohistochemistry both in villous and non-villous trophoblast cells. Furthermore, XOR enzyme activity was also detected through the radiochemical method, although XDH/XO activity in the placenta was much lower than in the liver [50].

### 4.2. Methods of Assessment of XO Activity

It is important to note that there are various methods to assess XDH/XO activity in bio samples, and not all the approaches have the same sensitivity, such as spectrophotometry, fluorometry (FL), colorimetry, and radiometry [51]. The radiochemical assay is 10 times more sensitive than the spectrophotometric assay. The radiochemical assay uses a radioactive marker to evaluate the conversion of [14C] xanthine or [14C] hypoxanthine (contained in the reaction mixture) to [14C] uric acid, while the spectrophotometric assay measures the formation of uric acid from xanthine [50,51]. Several studies also employ a combination of high-performance liquid chromatography and ultraviolet detection (LC/UV). This method uses xanthine as a substrate; LC allows the separation of the produced uric acid, while UV detectors measure UA production. Other variants include LC/FL, which utilizes fluorescence detection, and LC/HRMS (high-resolution mass spectrometry), which uses mass spectrometry [51]. A study conducted at the University of Bologna by Battelli et al. recommends performing the evaluation of XDH/XO enzymatic activity using an ELISA method, demonstrating that it is more sensitive than the spectrophotometric method and easier to be implemented in routine clinical analyses compared to other sensitive tests (radiochemical, high-performance liquid chromatography, or FL methods). However, ELISA assays compared to enzyme activity-based assays, detect both active and inactive enzyme molecules [52]. Immunohistochemistry and XO plasma concentrations can help identify the level of XO expression in tissue but they do not provide precise information on XO activity since they detect both the active and inactive forms of the enzyme. For this reason, 12 out of the 14 included studies evaluated XO activity, while 1 study (Shannon A. Bainbridge et al., 2009 [27]) assessed only immunohistochemical expression, and 1 study (Ramya Rajshekar et al., 2021 [34]) measured only XO plasma levels.

Most of the studies included in the systematic review evaluating XO activity used the spectrophotometric method. Only the study by Oguz Elmas et al. [31] used a more sensitive method, namely high-performance LC, whereas another included study indirectly estimated XO activity as the ratio of two urinary metabolites of caffeine (1 MX, the substrate of XO, and 1 MU, the product of XO), measured with the high-pressure LC method in urine samples after caffeine administration. As XO is also involved in the metabolism of methylxanthines, the caffeine metabolic ratio 1 MU/(1 MX + 1 MU) has been shown to be a specific indicator of in vivo XO activity [53]. Overall, XO activity was assessed in maternal serum, cord blood, and placental samples (after homogenization and centrifugation). Furthermore, we emphasize that only one study compared the proportions of the enzyme in its different isoforms by evaluating [14C] uric acid production in the presence (XDH) and absence (XO) of NAD (A. Many et al., 2000 [21]). All other studies evaluated only XO, as it is the XOR isoform that primarily contributes to ROS production, promoting OS and, consequently, the development of numerous pathological conditions.

### 4.3. Role of XO in ROS Production: The Ischemia Reperfusion Mechanism

Although XOR is constitutively present in its dehydrogenase form, various conditions can promote the reversible conversion of NAD+-dependent XDH into the oxidase form through sulfhydryl group oxidation, or irreversibly through limited proteolysis [54]. In particular, many studies have focused on the role of the ischemia–reperfusion (H/R) mechanism in increasing XO activity and expression. Ischemia first induces Ca++-dependent proteolytic conversion of XDH to XO and increases levels of its substrates (xanthine and hypoxanthine). Then, reperfusion provides oxygen to the XO enzyme, leading to ROS production, which causes cytotoxicity and tissue damage [55]. In the reaction catalyzed by the oxidase form, oxygen acts as the electron acceptor, producing superoxide ions through a one-electron reduction and generating hydrogen peroxide via a two-electron reduction [43]. Moreover, hypoxia and inflammatory cytokines (TNF-α, IL-1β, IFN-γ) induce XDH expression in tissues and vascular endothelial cells, from which it is released into the circulation. Circulating XDH is quickly converted to XO, which then avidly binds to the negatively charged glycosaminoglycans (GAGs) on the apical surface of vascular endothelial cells, amplifying local ROS concentrations [56]. Oxygen tension influences XOR activity not only by regulating the gene expression of the enzyme but also through post-translational regulation, including phosphorylation and sulfurization of XOR molecules. For instance, ischemia increases mRNA expression (leading to an increased amount of XOR proteins) via pre-translational regulation, while also boosting XO activity by promoting the phosphorylation of XOR molecules.

It is also worth mentioning that, contrary to previous beliefs, the XDH isoform can also produce ROS. When NAD+ is abundantly available, the generation of O^2−^ by XDH is limited. However, under ischemic conditions, XDH operates as an oxidase of NADH and generates superoxide radicals by transferring an electron from NADH to FAD and reducing molecular oxygen. Nevertheless, our review has focused on the role of XO in ROS production and tissue damage [43].

### 4.4. The Ischemia Reperfusion Mechanism: Human Placental In Vitro Studies

OS is implicated in the pathogenesis of various obstetric pathologies, including HDP [57,58,59,60,61,62], early miscarriage [60,62,63], GD [64,65,66], preterm delivery [67], and fetal growth restriction [68]. Several in vitro studies have been conducted to determine if the H/R pathway could be one of the mechanisms responsible for the increase in OS observed in diseased placental tissue. It has been shown that several changes observed in placental tissue during in vitro altered perfusion are similar to those reported in placentae from certain pregnancy pathologies, suggesting that H/R may be one of the underlying pathophysiological mechanisms of many obstetric conditions. In 2001, Hung T. and colleagues examined the oxidative status of human placental samples in vitro during periods of hypoxia and reoxygenation (H/R model). Using a fluorogenic probe, they detected a high generation of ROS in the villous endothelium and, to a lesser extent, in the syncytiotrophoblast and stromal cells when hypoxic tissues were reoxygenated [69]. The resulting OS stimulated apoptosis in human placental tissues since H/R could induce the release of cytochrome c from mitochondria and the activation of caspases in the syncytiotrophoblast and fetal endothelial cells [70]. Another in vitro model that allows for the analysis of H/R-induced increase in OS is the dual-perfused placental tissue model. This model compares the perfusion of an isolated cotyledon of term placenta using a standard medium and a medium containing xanthine plus XO, which generates ROS [71]. This model demonstrated that the inflammatory response following exposure to the X + XO medium is responsible for the immunohistochemical staining of IL-1b in the villous stromal cells, an increase in various inflammatory cytokines (TNF-alpha, IL-1beta, IL-6, IL-8, and IL-10) in the maternal compartment, accumulation of 8-isoPGF2alpha (a marker of lipid peroxidation) in the fetal compartment, and shedding in the maternal compartment, as shown via flow cytometry [71]. Additionally, a study conducted by Murata et al. demonstrated that incubating a cell culture of extravillous trophoblast (EVT) cells with X + XO induced ROS production by XO, which in turn triggered apoptosis and altered EVT functions, including invasion, tube-like formation, and differentiation [72].

### 4.5. Role of XO in Obstetric Pathologies: Discussion of Studies Included in the Review

After clarifying the role of XO and ROS production in the placenta, as well as the association between OS and placental diseases, we now delve deeper into the specific role of XO in individual obstetric pathologies by analyzing the studies included in this systematic review.

#### 4.5.1. XO and Hypertensive Disorders of Pregnancy

Hypertensive disorders, including PE and gestational hypertension, are common pregnancy complications and represent major causes of maternal and fetal morbidity and mortality worldwide [73]. Pathogenetically, the historical hypothesis sees various pathogenetic mechanisms that occur at two stages: abnormal placentation early in the first trimester, followed by a “maternal syndrome” characterized by systemic vascular dysfunction in the later second and third trimesters. Various pathophysiologic mechanisms have been proposed for the placental dysfunction observed in stage 1, including OS. Specifically, inadequate spiral arteriolar remodeling by trophoblasts leads to narrow maternal vessels and relative placental ischemia. Intermittent hypoxia and reoxygenation caused by poor spiral artery invasion may result in OS, which promotes the transcription of antiangiogenic factors such as the soluble form of VEGFR-1 (sFLT1), leading to systemic vascular dysfunction that is also responsible for long-term cardiovascular impairment [74,75,76]. ROS can arise from various sources, such as mitochondrial stress or increased XO expression and activity caused by ischemia, as previously described [77]. The association between XO activity and HDP has been assessed in 9 of the 14 studies included in this systematic review.

The role of XO in PE was first investigated in a histological study by Many et al. in 2000 [21]. In this study, 10 placental specimens obtained from preeclamptic women were compared with 12 placental specimens from uncomplicated pregnancies. First, the immunohistochemical expression of XO was analyzed. In control samples, no XO expression in chorionic villi was found, whereas in PE samples, immunoreactivity was dramatically increased in the trophoblast layers, the stromal cores of blood vessels, and the invasive cytotrophoblasts. Furthermore, XDH/XO holoenzyme activity was compared with XO isoenzyme activity in placental villi and placental bed curettings from both cases and controls using a radiochemical method, which is one of the most sensitive methods for assessing XO activity. No difference in XDH/XO activity and XO activity was detected in placental villi samples between the PE and control groups. However, XDH/XO activity and XO activity in placental bed curettings (which contain invasive cytotrophoblasts) were significantly higher in the PE group compared with the control group. The invasive cytotrophoblast, with oxygenation determined by non-anastomosing basal arteries and diffusion through several cell layers of decidua, is more susceptible to hypoxia and, consequently, to increased XO activity, whereas the villous cytotrophoblast extensively exposed to intervillous blood flow is less subject to hypoxic stimulus. Despite similar XO activity in villous trophoblasts between groups, an increase in staining for nitrotyrosine was found in these cells, which can be considered an indirect marker of OS and endothelial dysfunction. Indeed, NO interacts with increased exposure to superoxide to form peroxynitrite, which causes nitration of tyrosine residues on proteins, forming nitrotyrosine. The resulting depletion of NO was assessed in the study included in the review conducted by V. Bambrana et al. [29], showing a decrease in NO in PE compared to healthy pregnant women before delivery. Therefore, the production of ROS, which interferes with the activity of NO synthase and the availability of NO, contributes to endothelial damage and reduced endothelial NO-mediated vasodilation, creating an altered vasomotor tone, as documented in some clinical studies in women with PE [78]. Finally, peroxynitrite, which belongs to reactive nitrogen species (RNS), can react with molecules such as proteins, DNA, and RNA, altering their structure and function [1]. Another histological study conducted by Shannon A. Bainbridge et al., 2009 [27] demonstrated that the increase in XO activity is not limited to certain trophoblast subpopulations but also affects the skin of PE women. The authors found intense XO immunoreactivity within the stratum granulosum layer of the epidermis of skin biopsies collected from PE patients. The same result was observed in biopsies from patients with inflammatory conditions in active states (systemic lupus erythematosus, dermatitis, lichen simplex, mixed connective tissue disease, and bullous pemphigoid) [27]. Inflammatory cytokines such as TNF-alpha, IL-1beta, and IFN-gamma induced an increase in XO activity by 2, 2.5, and 8 times, respectively. This study emphasizes the importance of inflammation in PE, providing another potential factor contributing to the OS-induced progression of PE. XO overexpression in PE may be due not only to H/R mechanisms but also to inflammatory conditions caused by immune dysregulation and the release of apoptotic trophoblast cell residues into circulation [78]. In fact, in vitro studies have shown that elastase released by activated neutrophils induces the conversion of XDH to XO in endothelial cells, contributing to systemic OS [79]. In this regard, Ramya Rajshekar et al., 2021 [34] conducted a case–control study evaluating XO and plasma elastase levels in 30 PE patients and 30 normotensive pregnant individuals. In addition to confirming that XO levels were significantly higher in PE patients compared to controls, the authors demonstrated that elastase was 4.5-fold higher in PE patients than in controls. The difference in mean elastase levels was not statistically significant. Moreover, XO levels were positively correlated with elastase levels, confirming the role of leukocyte-induced activation in increasing XO activity in women with PE. As PE is a systemic condition, Abdulkadir Yildirim et al., 2004 [23] measured serum XO activity in 25 women with PE and 15 healthy pregnancies to assess whether the increased XO activity at the placental level in PE was also associated with increased systemic levels. The study found that mean plasma XO activity was higher in the PE group than in the healthy pregnancy group, confirming the systemic rather than local action of XO and OS. Moreover, this study also found a correlation between the severity of PE and the level of XO serum activity: XO activity was significantly higher in severe PE compared to mild PE. These findings were later confirmed in the study by Oguz Elmas et al., 2016 [31], which found that XO activity in 20 women with PE was higher than in controls and had strong correlations with blood pressure (diastolic, systolic, and mean arterial pressure), suggesting that XO activity increases proportionally with the severity of the condition (assessed in terms of blood pressure values). Additionally, according to ROC analysis, the authors found that the predictive values of XO activity and UA levels were significantly higher than those of allantoin. The study by V. Bambrana et al., 2015 [29] also assessed the role of XO in PE. The authors measured XO activity using a spectrophotometric method both during the antenatal and postpartum periods in 50 normal pregnancies and 50 PE pregnancies to determine if measuring XO is an accurate predictive marker for PE. They found that plasma XO activity in the PE group was 5.26 times greater than in normotensive pregnant women before delivery. Plasma XO activity decreased significantly after delivery in both PE and control groups but remained significantly greater (2.1 times) in the PE group vs. healthy controls (*p* < 0.001). In 2005 Aysun Bay Karabulut et al., 2005 [24] investigated the hypothesis that OS extends beyond the maternal compartment to the fetus. The authors analyzed not only maternal serum XO activity but also fetal plasma XO activity and MDA levels (a marker of lipid peroxidation) in maternal and cord plasma, finding that these parameters were also elevated in fetuses of PE pregnancies. Similarly, the study by M. Bogavac et al., 2012 [28] examined fetal conditions in PE pregnancies by measuring XO levels and activity in amniotic fluid samples from 66 normal pregnancies and 23 pregnancies complicated by gestational hypertension. The authors found a statistically significant difference in XO concentration and activity between the two study groups. Finally, Ilona Németh et al., 2002 [22] compared a group of healthy women with a group of pregnant women with gestational hypertension without signs of renal impairment, finding increased XO activity in hypertensive subjects. However, it is worth noting that in this study, XO activity was not measured in serum or placental lysates but was estimated indirectly as a ratio of urinary metabolites of caffeine after caffeine administration, as XO is involved in the metabolism of methylxanthines. Two included studies (Abdulkadir Yildirim et al., 2004 [23] and Oguz Elmas et al., 2016 [31]) also analyzed XO activity alongside uric acid levels, which were found to be increased in PE women compared to controls. Some authors argue that the hyperuricemia observed in PE women is merely a secondary epiphenomenon due to reduced UA excretion caused by renal damage and increased tubular reabsorption due to hypovolemia, both characteristic of PE [80]. However, it could also result from increased XO activity, as it is the terminal product of purine metabolism. This hypothesis is corroborated by the evidence that serum uric acid to serum creatinine ratio, which normalizes uric acid for kidney function allowing for an indirect selection of patients with hyperactivation of XO, is elevated in women with PE and associated with adverse pregnancy outcomes [81]. Furthermore, hyperuricemia in PE patients may also play a pathogenetic role, as demonstrated by some in vitro studies showing that uric acid reduces trophoblast invasion into maternal uterine vessels, and murine models of hyperuricemia develop a PE-like syndrome during pregnancy. Finally, several observational studies found a correlation between hyperuricemia and the development of HDP [82,83,84]. Four included case–control studies (Abdulkadir Yildirim, 2004 [23]; Bogavac et al., 2012 [28]; Min Shang et al., 2015 [30]; Ebru Biberoglu et al., 2016 [32]) evaluated not only XO activity but also antioxidant protein levels in serum. Specifically, serum levels of SOD were found to be decreased in PE women compared to controls, likely due to consumption by excess of ROS. Also, at the placental level, immunohistochemical expression of SOD is reduced, as demonstrated by the histological study conducted by Many et al., 2000 [21].

#### 4.5.2. XO and Gestational Diabetes

GD is the most common pregnancy complication, characterized by the onset of glucose intolerance during pregnancy. It affects about 10–15% of pregnancies and can lead to fetal macrosomia, prenatal mortality, and an increased long-term risk of developing type 2 diabetes mellitus [85,86]. Low-grade chronic inflammation and OS play central roles in the pathophysiology of GD. Risk factors for GD, such as obesity, are associated with increased numbers of resident adipose tissue macrophages that secrete pro-inflammatory cytokines, including TNF-α, IL-6, and IL-1β. This inflammatory state impairs insulin signaling and inhibits insulin release from β-cells [65]. Moreover, hyperglycemia induces OS through several metabolic mechanisms. Increased XO activity is also a key factor in this process [66]. Excessive ROS production inhibits insulin-stimulated glucose uptake by interfering with GLUT4 expression, contributing to the onset of GD [87]. These two pathophysiological mechanisms—low-grade inflammation and OS—are interconnected. Inflammatory cytokines stimulate XO activity and the conversion of XDH to XO, leading to increased superoxide radical production [56]. The association between GD and OS has also been demonstrated in animal studies, confirming the significant role of ROS in the onset of GD. For example, in a study using streptozotocin-induced diabetic pregnant rats, there was a depletion of the antioxidant defense system with decreased SOD and GSH activity and increased uric acid levels [88]. Despite the streptozotocin animal model (also referred to as a chemical model) being able to induce streptozotocin-induced fetal malformations [89], several authors have demonstrated that fetal malformations are due to OS and are clearly dependent on the embryonic levels/activity of antioxidant enzymes in genetic models of diabetes [66]. Of key importance is the immaturity of the fetal antioxidant system, making it particularly susceptible to the damaging effects of OS [66]. Indeed, there is evidence that maternal diabetes during pregnancy can induce OS in the fetus. For instance, a study by A. Biri et al., 2006 [25] investigated OS markers in the placenta, maternal plasma, and cord plasma in 13 pregnant women with GD and 13 women with normal glucose tolerance. They found significantly increased XO activity in maternal plasma and placental tissues in the GD group compared to controls, confirming the key role of XO in the pathophysiology of this disease. Higher levels of active XO were also detected in umbilical cord blood from fetuses of GD pregnancies, suggesting fetal ROS formation in this condition. The study by M. Bogavac et al., 2012 [28] also demonstrated increased fetal XO activity in amniotic fluid samples from 18 women with GD and 66 women with uncomplicated pregnancies. Min Shang et al. assessed XO activity in maternal plasma, cord plasma, and placenta samples in two studies, one published in 2015 [30] and another in 2018 [33]. The 2015 study compared oxidative and antioxidant status in GD women diagnosed by the International Association of Diabetes and Pregnancy Study Groups (IADPSG) criteria (requiring only a glucose value higher than the defined cutoffs during a 75 g 2 h oral glucose tolerance test) versus American Diabetes Association (ADA) criteria (requiring two or more glucose values above the cutoffs). They found OS in both subgroups, but women diagnosed by ADA criteria had higher maternal XO activity and, consequently, greater OS compared to those diagnosed by IADPSG criteria. This suggests that ADA criteria may identify more severe cases with poorer glycemic control. The study also found significant positive correlations between cord and placental XO levels and HbA1c values, further corroborating the hypothesis that women with poor glycemic control had higher levels of XO-induced OS. In the 2018 study, the authors evaluated the association between XO activity and the severity of GD by assessing markers of insulin resistance and sensitivity (HOMA-IR and QUICKI index, respectively). They found that women with GD had higher HOMA-IR and lower QUICKI compared to controls, indicating more severe insulin resistance. OS markers in maternal plasma, cord plasma, and placenta were positively correlated with HOMA-IR and negatively correlated with QUICKI, demonstrating that OS correlates with the severity of the condition. Additionally, levels of adipokines involved in insulin resistance were also positively correlated with XO activity, suggesting a role for OS in the pathogenesis of insulin resistance. Both studies by Min Shang et al. found that XO activity in maternal and cord plasma was negatively correlated with newborn birthweight, and macrosomic fetuses had increased XO levels in cord plasma, suggesting the neonatal impact of increased maternal XO and OS during pregnancy. Including XO measurements in the management of women with GD could not only improve the predictive and prognostic accuracy in this condition but also provide insights into the future cardiovascular risk of both the mother and the offspring.

#### 4.5.3. XO and Intrauterine Growth Restriction

IUGR is defined as an estimated fetal weight below the tenth percentile for gestational age, typically arising in the second trimester of pregnancy. While the term “small for gestational age” (SGA) may encompass constitutionally small but healthy fetuses, IUGR signifies a pathological intrauterine growth retardation [90]. The causes of IUGR can be classified as fetal (e.g., chromosomal abnormalities, infections), maternal (e.g., nutritional deficiencies), or placental factors. Notably, uteroplacental dysfunction, such as abnormalities in uteroplacental blood vessels, accounts for 80% of IUGR cases [90].

Adequate extravillous trophoblast invasion and rapid villous angiogenesis are crucial for proper placental development and subsequent fetal growth. Altered maternal arterial remodeling has been linked to the pathophysiology of obstetric syndromes like IUGR through placental malperfusion. A key feature of the placenta in IUGR cases is the reduced volume, surface area, and vascularization of intermediate and terminal villi [91]. Inadequate development of spiral arteries impairs nutrient transport to the fetus and increases resistance within the umbilical circulation, leading to H/R, which exacerbates OS and damages placental tissue [37]. Several studies have reported increased OS markers in maternal and umbilical cord plasma, and placental tissues in pregnancies complicated by IUGR, confirming that OS plays a role in the condition [90]. An interesting study by A. Karowicz-Bilińska [90] demonstrated that the administration of arginine (a precursor of NO) and acetylsalicylic acid (an inhibitor of thromboxane synthase) reduced OS markers in IUGR pregnancies. These substances decrease thromboxane, which is involved in the increased generation of ROS. However, this study did not assess fetal weight and did not use XO as a marker of OS. In turn, XO activity could play a crucial role in the OS that characterizes this condition. A. Biri et al., 2007 [26] conducted a case–control study involving 13 singleton pregnancies complicated by IUGR and 12 healthy singleton pregnancies. Authors investigated OS in women with IUGR compared to those with normal fetuses and found a significant increase in XO activity in the IUGR group in maternal and umbilical cord plasma, and placental biopsies. Another study (Ebru Biberoglu et al. [32]) published in 2016 assessed the relationship between IUGR and OS by comparing XO activity simultaneously in the circulation and myometrium. However, this study did not confirm the hypothesis of increased XO in IUGR pregnancies. The researchers found that serum and myometrial XO activity were comparable between the IUGR and control groups. Nevertheless, the authors observed higher malondialdehyde (MDA) concentrations, another marker of OS, in the serum and lower concentrations in the myometrial samples of the IUGR group compared to the control group. This suggests a “spillover” of MDA from the uterus to the circulation protecting the fetus from ROS. The conflicting results regarding OS markers in these studies could be attributed to the multifactorial nature of fetal growth restriction, which involves maternal, fetal, and placental pathologies. Additionally, potential biases, such as the inclusion of pregnancies with fetal weights below the tenth percentile but without growth restriction, may have influenced the outcomes.

### 4.6. Strategies for the Inhibition of Xanthine Oxidase during Pregnancy

Despite the fact that XO inhibitor drugs have been widely used for many years in the treatment of gout, there are no conclusive data regarding their safety during pregnancy.

Since risk cannot be ruled out, both allopurinol and febuxostat are currently classified as category C drugs, according to the original five-tier letter system of the Food and Drug Administration (FDA). According to the new FDA classification, the “Pregnancy and Lactation Labeling Rule” (PLLR), a more comprehensive narrative labeling system, allopurinol, and febuxostat belong to the group of drugs without well-controlled studies conducted in humans and/or adverse fetal effects in animal studies [92].

Regarding febuxostat, no adverse developmental effects and no teratogenicity were observed in embryo–fetal development studies, with oral administration to pregnant rats and rabbits during organogenesis at doses that produced maternal exposures up to 40 and 51 times, respectively, the exposure at the maximum recommended human dose (MRHD) [93]. As for allopurinol, the use of this drug in animal studies has induced species-specific reproductive toxicity. In humans, congenital malformations have been reported in some cases of newborns exposed to allopurinol. However, according to a systematic review from 2018 and another from 2024, the association between allopurinol and teratogenicity appears to be weak and with uncertain causality, and the currently available data are insufficient to make a definitive judgment [94,95].

To overcome the potential risks associated with the treatment of pregnant women with XO inhibitors, the incorporation of some foods with direct antioxidant activity, such as polyphenols, may be of help in pregnancy complications associated with an increased level and/or activity of XO. Several plant-derived bioactive compounds (luteolin, quercetin, isorhamnetin, galangin, prosapogenin, hesperetin, and theaflavin-3,3′-digallate) and dietary cranberry juice, purple grape juice, and black tea may inhibit XO activity [96,97].

Additionally, recent evidence suggests a protective role of certain bacteria isolated from fermented foods, which can prevent hyperuricemia by inhibiting purine absorption and XO activity [98]. It may also be beneficial to reduce the intake of certain foods, such as orange juice and pink grapefruit juice, which directly stimulate XO activity [97], as well as high purine-rich foods (such as animal meats, fish, organs like liver and fish milt, and yeast) that provide a large amount of substrate to the XO enzyme [99,100].

Finally, it must be considered that some medications commonly prescribed for HDP also have the pleiotropic effect of reducing OS through XO inhibition and/or the inhibition of free radicals production. For example, it has been demonstrated that certain beta-blockers [101,102], calcium channel blockers [103,104], magnesium sulfate [105], and acetylsalicylic acid [106] exhibit antioxidant properties.

This review has some limitations: firstly, XO was evaluated in different tissues and measured using different analytical methods in the included studies. Secondly, the total number of included studies was low, with a low to moderate sample size in each study. Finally, patients were not stratified by age and gestational period.

## 5. Conclusions

The primary objective of this systematic review was to evaluate the existing studies on the associations between XO levels, activity, and/or expression at the fetal, maternal, and placental level, and major pregnancy pathologies such as HDP, GD, IUGR, PTB, and RPL.

The rationale for this research stems from previous studies demonstrating that OS is a key component of the pathophysiology of most frequent obstetric diseases and XO is an enzymatic marker of OS. Pregnancy-related pathological conditions are linked to altered placentation, leading to H/R processes. This, in turn, triggers the reversible or irreversible conversion of the XDH isoform to the XO isoform, which becomes an important source of ROS (especially superoxide and hydrogen peroxide), resulting in cellular and tissue damage. Moreover, H/R promotes apoptosis of trophoblast cells, leading to the shedding of cellular fragments into circulation, further exacerbating inflammation. Inflammatory cytokines also increase XO activity and expression, creating a vicious cycle. Major pregnancy complications such as PE and GD are also associated with an increased risk of future cardiovascular disease; studies outside pregnancy have demonstrated that XO and its terminal product uric acid are independently associated with cardiovascular risk. We therefore believe that, unlike other OS markers, assessing XO levels and activity could provide valuable information not only for pregnancy outcomes but also for the future risk of developing cardiovascular diseases later in life. Furthermore, since XO inhibitors are already used to treat symptomatic hyperuricemia, these medications may demonstrate in the future a cardioprotective role also in women with a history of pregnancy complications. Further studies are needed to evaluate the short- and long-term outcomes of patients with elevated XO activity during pregnancy. To the best of our knowledge, there are no studies that have investigated the correlation between XO levels in pregnant patients with the development of cardiovascular disease later in life. Additionally, no longitudinal studies have been conducted to monitor XO levels throughout pregnancy and after delivery, to identify a potential predictive role in disease onset, beyond its pathophysiological role.

All the studies but one (on women with IUGR fetuses) included in the present systematic review have demonstrated increased XO levels, activity, and/or expression in women diagnosed with HDP, GD, and IUGR. Moreover, the increase in XO is often systemic, affecting not only the placenta but also the entire maternal vascular system, and extending to the fetus, increasing the risk of fetal pathologies. However, no study has evaluated a potential association between XO and RPL or PTB.

Further evidence on the role of XO in pregnancy outcomes would not only deepen our understanding of the pathophysiological mechanisms at the basis of pregnancy complications but also potentially open new pharmacological possibilities for the prevention or treatment of obstetric pathologies characterized by increased XO activity. This would also contribute to the prevention of fetal health issues, as the fetus is exposed to maternal ROS and XO.

## Figures and Tables

**Table 1:** Table summarizing the content of the 14 included studies.

Author, Year	Studied Population	Methods of XO Measurement	Results
A. Many et al., 2000 [21]	Histological study: 12 control placental specimens obtained from HPC vs. 10 placental specimens obtained from PE women.	Examination of XDH/XO holoenzyme activity and XO isoenzyme activity in placental villi and placental bed curettings by radiochemical method, adding xanthine and evaluating NAD production. Expression of XDH/XO in tissue samples by immunohistochemical staining.	In control samples, no XO expression in chorionic villi was found; a relatively weak XO immunoreactivity was found only in syncytiotrophoblasts. In PE samples, immunoreactivity dramatically increased, associated not only with the trophoblast, but also with the stromal cores of the blood vessels. Also, invasive cytotrophoblasts manifest increased expression of XO enzyme in PE. No difference in XDH/XO activity and XO activity was detected in placental villi. In placental bed curettings XO activity was significantly higher in the PE group compared with the control group.
Ilona Németh et al., 2002 [22]	Case–control study: 16 pregnant women with gestational hypertension vs. 14 HPC (matched for maternal age, parity, and gestational age) vs. 15 HC (matched for age)	After administration of a dose of caffeine (10 mg/kg), XO activity was estimated as ratio of two urinary metabolites of caffeine, 1 MX, the substrate of XO, and 1 MU, the product of XO measured by high-pressure liquid chromatographic method. Urine was collected over a 6 h period after caffeine administration.	A significant decrease in the urinary concentration of 1 MX was shown in the hypertensive subjects without any decrease in the urinary concentration of 1 MU. As a result, the XO activity index rose significantly in this group. Increased levels of UA were shown in hypertensive individuals. Other markers of OS, such as lipid peroxidation products, were more elevated in hypertensive patients compared to both HC and HPC. The XO activity index was higher in pregnant women with hypertension (0.849 ± 0.096) than in HPC in ed HC (0.596 ± 0.105 *p* < 0.01; 0.542 ± 0.049 *p* < 0.01, respectively).
Abdulkadir Yildirim et al., 2004 [23]	Case–control study: 25 PE women (17 mild PE and 8 severe PE) vs. 15 HPC vs. 15 HC.	Plasma XO activity was assayed spectrophotometrically.	Mean plasma XO activity was higher in both mild (*p* < 0.05) and severe (*p* < 0.001) PE groups than in the HPC group, with PE severity defined by blood pressure values. Other markers of OS showed a reduction in antioxidant systems (SOD, GSH-Px) in PE compared to both HPC and HC groups. In the PE group, SBP and DBP had a significant positive correlation with plasma XO activity (r = 0.59, *p* < 0.01 and r = 0.68, *p* < 0.001, respectively).
Aysun Bay Karabulut et al., 2005 [24]	Case–control study: 29 PE singleton pregnancies vs. 33 HPC (matched for maternal age and gestational age).	Plasma XO activity was assessed in maternal and cord blood by spectrophotometric methods. Maternal blood was drawn from the antecubital vein immediately after delivery.	XO and ADA activities and MDA levels in maternal and fetal plasma were significantly higher in the PE group than in the controls (*p* < 0.05).
A. Biri et al., 2006 [25]	Case–control study: 13 singleton pregnancies complicated by GD vs. 13 singleton HPC (matched on gestational age, maternal age, and BMI).	XO assessment was performed In maternal and cord plasma, and placental tissue samples (both collected at the time of Cesarean section). XO activity was analyzed spectrophotometrically.	XO activity of the GD group was significantly higher in maternal plasma (*p* < 0.05), cord plasma (*p* < 0.05), and placental tissues (*p* < 0.005), compared to the control group. Higher levels of HbA1c were correlated with lower levels of AOP (r = −0.419; *p* < 0.05) and MDA (r = −0.348; *p* < 0.05) in cord blood, and higher levels of MDA in maternal serum (r = 0.405; *p* < 0.05), which means that OS in maternal blood is associated with diabetes progression.
A. Biri et al., 2007 [26]	Case–control study: 13 pregnancies complicated by IUGR vs. 12 singleton HPC (matched on gestational age, maternal age, and BMI). The fetuses with a fetal birthweight < 10th percentile for gestational age were defined as having IUGR.	In the cord and maternal plasma and placenta tissue samples (both collected at the time of Cesarean section) XO activity was assayed spectrophotometrically.	Maternal serum XO activity was significantly higher in the IUGR group than in the control group (1.251 ± 0.674 mIU/mL vs. 0.20 ± 0.019 mIU/mL; *p* < 0.0005). Cord plasma levels of XO were also significantly higher in the IUGR group (1.97 ± 0.73 mIU/mL vs. 0.237 ± 0.143; mIU/mL, *p* < 0.0005). Placental levels of XO were significantly higher in the IUGR group than in the control group (0.023 ± 0.001 mIU/mL vs. 0.12 ± 0.004 mIU/mL; *p* < 0.025). Other redox metabolites showed a similar trend between the two groups, except for plasma levels of CAT. Placental tissue XO (*p* < 0.005) and other markers levels were significantly higher, while the CAT level (*p* < 0.005) was lower in the IUGR group.
Shannon A. Bainbridge et al., 2009 [27]	Case–control study: 5 PE vs. 5 HPC (matched for age, pre-pregnancy BMI) vs. 6 HC vs. 15 nonpregnant with inflammatory disease (5 with systemic lupus erythematosus, 3 with dermatitis, 1 with lichen simplex, 1 with mixed connective tissue disease, 5 with bullous pemphigoid).	Expression of XO was assessed by immunohistochemical fluorescent staining of skin biopsies. In all the pregnant women, biopsies were obtained from the cesarean section skin incision. In the nonpregnant participants with chronic inflammatory disorders, the biopsies were collected from lesional skin. In the HC, the skin biopsies were collected from benign skin lesions.	PE skin biopsies demonstrated intense XO immunoreactivity within the stratum granulosum layer of the epidermis. Faint and sporadic XO-positive staining was observed in the stratum granulosum layer of the epidermis in all the healthy pregnant control biopsies. All skin biopsies collected from nonpregnant patients with inflammatory conditions demonstrated immunoreactivity to XO within the stratum granulosum layer of the epidermis. Skin biopsies collected from HC demonstrated very little to no XO immunoreactivity.
M. Bogavac et al., 2012 [28]	Case–control study: 95 women with complicated pregnancy (54 with local infection, 18 GD, and 23 gestational hypertension) vs. 66 HPC.	Levels of XO were determined spectrophotometrically in amniotic fluid samples collected between 16 and 19 weeks of pregnancy during amniocentesis.	XO activity, though very low, was present in amniotic fluid samples and there was a statistically significant difference in XO activity between controls and all investigated study subgroups: patients with bacterial vaginosis (*p*-value < 0.015), patients with PIH (*p*-value < 0.001), and patients with GD (*p* < 0.001). Interestingly, other antioxidant enzyme systems showed no differences between groups.
V. Bambrana et al., 2015 [29]	Case–control study: 50 PE pregnancies vs. 50 HPC.	Plasma XO activity (measured with the spectrophotometric method was determined from samples during antenatal (at 30–39 weeks of gestation) and postpartum period.	The plasma XO activity was elevated in the PE group compared to HPC before (205 U/L ± 197.02; 39.10 U/L ± 54.04, respectively, *p* < 0.001) and after delivery (96.6 ± 141.3 U/L vs. 17.9 U/L ± 14.2; *p* < 0.001). The plasma XO activity registered during gestation decreased after delivery in both groups. Differences in NO were not significant. Pregnancy levels of SUA were higher in PE group.
Min Shang et al., 2015 [30]	A case–control study: 28 singleton GD vs. 40 HPC matched on gestational and maternal age, BMI, and parity.	Plasma XO levels were determined in maternal and cord plasma and placenta samples by spectrophotometric method.	The maternal, cord, and placental XO was significantly higher in GD women compared to HPC (*p*-value < 0.05). Other oxidative markers showed similar trends. Maternal plasma XO levels of GD vs. HPC were 15.75 ± 1.65 nmol/mL vs. 8.86 ± 1.97 nmol/mL, *p* < 0.05. Cord plasma XO levels were 14.13 ± 1.78 vs. 9.92 ± 2.20 nmol/mL, *p* < 0.05. Placental XO levels 51.18 ± 3.43 nmol/mL vs. 43.47 ± 8.02 nmol/mL, *p* < 0.05. Cord and placental XO had a significantly positive correlation with HbA1c values (R = 0.43 and R = 0.58, respectively). Several antioxidant status markers had a significant negative correlation with HbA1c values (*p* < 0.05). Increased XO levels in cord plasma were also found in macrosomia (*p* < 0.05).
Oguz Elmas et al., 2016 [31]	Case–control study: 20 PE pregnancies vs. 20 HPC (matched on gestational age and maternal age).	Plasma XO activity was assessed by high-performance liquid chromatography.	Serum XO activity was significantly higher in PE groups than in controls (0.49 ± 0.22 microM/min/L vs. 0.25 ± 0.13 microM/min/L; *p* < 0.0001). The XO activity, UA, and allantoin levels showed high correlations with blood pressure in the PE group (but not in the controls). Correlation between XO activity and blood pressure in PE group: r = 0.47 *p* = 0.039 for DBP; r = 0.37 *p* = 0.11 for SBP; r = 0.47 *p* = 0.038 for MAP. There was no statistically significant difference between XO activity and UA in terms of prediction of PE. However, XO activity had the highest predictive value. No differences were observed between groups in nitrite levels and their relationship with blood pressure values.
Ebru Biberoglu et al., 2016 [32]	Case–control study: 20 singleton pregnancies with an IUGR fetus vs. 20 singleton HPC (matched for mean age, BMI, and weight gain during pregnancy).	XO activity was measured spectrophotometrically in the myometrial lysates and maternal serum samples.	Serum XO was higher in IUGR vs. HPC (0.21 ± 0.04 mIU/L vs. 0.19 ± 0.02 mIU/L, *p* = 0.11), and myometrial XO lower (0.012 ± 0.01 mIU/mg; 0.013 ± 0.001 mIU/mg, *p* = 0.24), although differences were comparable. MDA and CAT concentrations were higher in the serum (*p* < 0.05) but lower in the myometrial samples (*p* < 0.01) of women with IUGR than HPC.
Min Shang et al., 2018 [33]	A case–control study: 105 pregnancies with GD vs. 103 HPC.	XO was measured in maternal plasma, cord plasma, and placenta samples with commercial kits by colorimetric methods using a spectrophotometer.	XO levels in maternal and cord plasma and placenta samples were significantly higher in women with GD (*p* < 0.05) compared to controls. The levels of markers of OS were altogether positively correlated with HOMA Index in maternal plasma, cord plasma, and placenta (r = 681 *p* < 0.001; r = 651 *p* < 0.001; r = 525 *p* = 0.072, respectively), whereas they were negatively correlated with QUICKI (r = −0.761 *p* < 0.001; r = −0.720 *p* < 0.001; r = −0.533 *p* < 0.001, respectively). All of the studied markers of OS in the macrosomic newborns were higher than in normal birthweight newborns and were negatively correlated with birthweight (*p* < 0.05).
Ramya Rajshekar et al., 2021 [34]	Case–control study: 30 women with PE Vs. 30 HPC matched for maternal age.	Plasma levels of XO were estimated spectrophotometrically.	XO level was significantly more elevated in PE (218.78 ± 220.42 U/L) than in controls (34.01 ± 38.26 U/L), *p* < 0.001. XO levels were positively correlated with elastase levels (r = 0.320; *p* < 0.05). Elastase was 4.5-fold more elevated in PE patients (26.81 ± 77.95 U/mL) than in controls (6.02 ± 3.4 U/mL), but the difference was not statistically significant (*p* > 0.05).

According to STROBE criteria, 5 articles obtained a score of 20 over 22 [22,25,27,30,34], 6 articles obtained a score of 19 over 22 [21,23,26,31,33], 1 article was scored 18/22 [24], 2 obtained a score of 16 over 22 [28,29]. List of abbreviations: ADA, Adenosine deaminase; AOP, antioxidant potential; BMI, Body Mass Index; CAT, catalase; DBP, Diastolic Blood Pressure; GD, gestational diabetes; GSH-Px, glutathione peroxidase; GSHR, glutathione reductase; GST, glutathione S-transferase; HC, healthy nonpregnant controls; HOMA-IR, homeostasis model assessment of insulin resistance; HPC, Healthy pregnant controls; HX, Hypoxanthine; IUGR, intrauterine growth restriction; LPO, lipid peroxides; MAP, mean blood pressure; MDA, malondialdehyde; NAD, nicotinamide adenine dinucleotide; NO, nitric oxide; SBP, Systolic Blood Pressure; PE, preeclampsia; QUICKI, quantitative insulin sensitivity check index; ROC, receiver operating characteristic; SOD, superoxide dismutase; TAC, total antioxidant capacity; UA, uric acid; X, xanthine; XDH, xanthine dehydrogenase; XO, xanthine oxidase; 1 MX, 1-methylxanthine; 1 MU, 1-methyluric acid; 8IsoP, 8-isoprostane.

## Data Availability

In the current study, no new data were generated.
